# The study protocol of the winners project: a randomized and controlled trial using a videogame-based training program in pediatric cancer survivors

**DOI:** 10.1192/j.eurpsy.2024.1344

**Published:** 2024-08-27

**Authors:** C. Gonzalez-Perez, E. Moran, N. Malpica, J. Alvarez-Linera, H. Melero, M. Alonso, M. Esteban, E. Fernández-Jiménez, A. Perez-Martinez

**Affiliations:** ^1^Children Psychology, Juegaterapia; ^2^Department of Pediatric Hemato-Oncology, La Paz University Hospital; ^3^ Medical Image Analysis and Biometrics Laboratory (LAIMBIO), Rey Juan Carlos University; ^4^Department of Neuroradiology, Hospital Ruber Internacional; ^5^Psychobiology and Methodology in Behavioral Sciences, Complutense University; ^6^Juegaterapia; ^7^Department of Psychiatry, Clinical Psychology and Mental Health, La Paz University Hospital, Madrid, Spain

## Abstract

**Introduction:**

Childhood cancer survivors have neurocognitive sequelae that in most survivor follow-up programs are underdiagnosed and for which there is usually no treatment plan.

Video games have demonstrated various psychological and neurocognitive benefits in different subpopulations, such as patients with organic neurological deficits or children with ADHD. However, few studies have been carried out using video games-based interventions in the paediatric oncology population.

**Objectives:**

The aim of this work is to present the WINNERS study protocol, the objectives of which are to diagnose the neurological and cognitive sequelae in child cancer survivors, and to demonstrate the benefit in these areas of a training program based on video games.

**Methods:**

A randomized controlled and unblinded trial is presented. Fifty-six patients aged 8 to 17 years stratified into two age groups (8-12 and 13-17) who had received any of the following treatments 1 to 6 years before the enrolment will be selected: high-dose chemotherapy with blood-brain barrier crossing, intrathecal or intraventricular chemotherapy, CNS radiotherapy or hematopoietic stem cell transplantation.

A neuropsychological evaluation will be performed consisting of a battery of neuropsychological tests to assess parameters such as attention, memory, visuospatial ability or speed of response, as well as a neuroimaging evaluation by structural and functional magnetic resonance imaging. The evaluation will be repeated 3 months and 6 months after the enrolment. Patients will be randomized to a treatment group or to a recycled waiting group. Intervention will consist on a 12-week training at home using 3 video games: a brain training game, an exergaming game and a skill training game.

**Results:**

According to the hypotheses of this study, it is expected that the proposed program of videogame-based interventions will improve neurocognitive and other wellbeing parameters in the intervention group.

**Conclusions:**

This study aims to improve the quality of care for patients who have survived a cancer disease by detecting sequelae that have so far been poorly attended, and by proposing a gamification-based intervention program that is effective and attractive for this population.

**Disclosure of Interest:**

None Declared

